# Exogenous Mitochondrial Pretreatment Enhances the Therapeutic Effect of UC-MSCs on NAFLD in Type 2 Diabetic Mice by Mediating Mitochondrial Transfer

**DOI:** 10.1155/sci/4639115

**Published:** 2025-08-25

**Authors:** Ruofan Hu, Jian Zhao, Yu Cheng, Wanlu Su, Rui Ren, Haixia Zhang, Yue Zhang, Anning Wang, Yiming Mu, Songyan Yu

**Affiliations:** ^1^Department of Endocrinology, Chinese People's Liberation Army General Hospital, No. 28 Fuxing Road, Beijing 100853, China; ^2^The 908th Hospital of Chinese People's Liberation Army Joint Logistic Support Force, Nanchang, China; ^3^Department of Endocrinology, Beijing Tiantan Hospital, Capital Medical University, Beijing 100070, China

## Abstract

**Background:** Nonalcoholic fatty liver disease (NAFLD) is the most prevalent form of chronic liver disease and is a comorbidity in type 2 diabetes (T2D) mellitus. Mesenchymal stem cell (MSC) is emerging as a potential therapeutic strategy for diabetes and NAFLD through mitochondrial transfer initiated by signaling from injured recipient cells. Thus, in this study, we investigated whether exogenous mitochondrial preconditioning of MSCs could exert superior effects on NAFLD and explore the role of MSCs-mediated mitochondrial transfer into hepatocyte.

**Methods:** After free HepG2 mitochondria pretreated, umbilical cord-derived MSCs (UC-MSCs) (mito-MSCs), T2D model mice were infused with equal amounts of MSCs/mito-MSCs via the tail vein once a week for 4 weeks. Body weight and random blood glucose were monitored weekly. After the end of treatment, the mitochondrial transfer level of MSCs before and after pretreatment were monitored by fluorescence tracing. Blood and liver were collected for biochemical and histopathological examinations. The number, morphology, and function of mitochondria in liver tissue were evaluated by tissue electron microscopy and western blot analysis. Real-time quantitative reverse transcription polymerase chain reaction (qRT-PCR) was performed to monitor the expression of genes associated with lipid metabolism and regulation pathways.

**Results:** Pretreatment of UC-MSCs enhanced the efficacy of MSCs in lowering blood glucose, liver transaminase, triglyceride levels, and reducing histological damage, which may be related to free mitochondria triggering autophagy of MSCs, which in turn promoted the entry of MSCs mitochondria into the liver tissue of diabetic mice.

**Conclusion:** Exogenous mitochondria could enhance the therapeutic efficacy of MSCs in NAFLD via mediating mitochondrial transfer, which offers a novel strategy for the improving the outcomes of MSCs cell-therapy for diabetes-related NAFLD.

## 1. Introduction

Diabetes is a major chronic disease that seriously threatens human health worldwide, with type 2 diabetes (T2D) accounting for more than 80% of all diabetes patients [1].Nonalcoholic fatty liver disease (NAFLD), a continuum of liver abnormalities from nonalcoholic fatty liver (NAFL) to nonalcoholic steatohepatitis (NASH), is one of the most common comorbidities of type 2 diabetes (T2D) mellitus accounting for 55% of the number of diabetic patients [2]. The pathogenic drivers of NAFLD are not entirely understood, but mitochondrial dysfunction has been proposed to play a significant role. Since, mitochondria are widely distributed in liver tissue [3], altered mitochondrial functionality contributes to fatty acid oxidation and oxidative phosphorylation damage, driving the important characteristics of human fatty liver disease, such as oxidative stress [4–6]. Similarly, mitochondrial morphological changes in NAFLD lead to inefficient oxygen use, ATP genertion, decreased total mtDNA, mqc-related genes, and MMP mRNA levels, and increased ROS levels in hepatocytes, leading to the progression of NAFLD [7, 8]. Previous studies have shown that interventions targeting liver mitochondria, such as mitochondria-targeted antioxidants [9, 10] and intravenous injection of exogenous functional liver mitochondria, can safely reverse NAFLD/NASH [11].. Therefore, repairing mitochondrial damage may also be an important therapeutic strategy for NAFLD.

Mesenchymal stem cells (MSCs) are a type of adult stem cells characterized by their multilineage differentiation potential [12]. Due to their capacity to facilitate tissue repair in various injuries, they have emerged as the preferred seed cells for cell therapy and regenerative medicine [13]. Human umbilical cord-derived MSCs (UC-MSCs), with their high proliferation potential and low allogeneic immunogenicity, are particularly suitable for clinical applications [14, 15]. In recent years, the therapeutic potential of UC-MSCs has been explored in NAFLD [16], effectively improving carbohydrate and lipid metabolism. However, our previous study has observed that the effect of MSC on T2D rats cannot be maintained for a long time [17, 18]. Consequently, enhancing the therapeutic effects of MSCs has become a prominent research focus in recent years.

Mitochondrial transfer is one of the paracrine mechanisms. It enables the transfer of healthy mitochondria to damaged cells via tunnel nanotubes (TNTs) [19], extracellular vesicles, gap junctions, and cell fusion. This process increases mitochondrial biogenesis, reestablishes aerobic respiration in target cells, and inhibits apoptosis [20]. Previous studies have shown that MSCs can transfer mitochondria to NAFLD-damaged liver cells and restore their function [7]. DAMPs are endogenous molecules released into the circulation or tissues by cell death and/or injury, which can act as “danger signals” to activate the immune system [21]. Mitochondria and their products (such as mitochondrial DNA and *N*-formyl polypeptides) can be recognized as DAMPs by specific receptors on immune cells and other cells, thereby triggering functional changes [22–24]. Mahrouf-Yorgov et al. [25]found that mitochondria from damaged cells, as DAMPs, triggered the induction of the protective enzyme heme oxygenase-1 (HO-1) in MSCs, stimulation of mitochondrial biosynthesis, and anti-apoptosis functions by using a coculture system consisting of MSCs and cardiomyocytes/endothelial cells. Based on the above research results, we hope to verify through in vivo and in vitro experiments that coculture of free mitochondria and UC-MSCs can enhance the therapeutic effect of UC-MSCs on liver injury in diabetic mice by increasing the transfer of healthy mitochondria in UC-MSCs to damaged liver cells.

## 2. Materials and Methods

### 2.1. Isolation, Culture, and Characterization of Human UC-MSCs

Human umbilical cords were obtained from women giving birth in the First Medical Center of PLA General Hospital. All of the subjects provided informed consent. The experimental protocols were approved by the Medical Ethics Committee of PLA General Hospital. Human UC-MSCs were isolated and cultured by previously described methods [26]. The cultured UC-MSCs at passage 3 were digested and harvested to identify the immunophenotype using FITC-conjugated anti-CD45, anti-CD90, and anti- HLA-DR; PE-conjugated anti-CD105 and anti-CD73; and PerCP-conjugated anti-CD34 antibodies byflow cytometry analysis. All the antibodies used for surface marker analysis were purchased from BD Company. The potential of UC-MSCs to differentiate into osteoblasts and adipocytes was verified as previously described [27].

### 2.2. Isolation and Delivery of HepG2 Mitochondria to UC-MSCs

The HepG2 cell line obtained from the Procell (WuHan, China), were routinely cultured in high-glucose DMEM supplemented with 10% heat-inactivated FBS, 100 U/mL penicillin, 100 lg/mL streptomycin, and 1% nonessential amino acid (NEAA). HepG2-derived mitochondria were isolated with the Mitochondria Isolation Kit for Cultured Cells (ThermoFisher Scientific, USA, Cat#89874) according to the manufacturer's instructions. Mitochondria were isolated under sterile conditions at 4°C. Mitochondrial protein concentrations were measured using the Detergent Compatible Bradford Protein Assay Kit (Beyotime, China, Cat#P0006C). To confirm the lack of living cells in the mitochondria suspension, we incubated the suspension in a plastic dish in an incubator 37°C. Under these conditions, no cells we found to adhere after several days of culture. UC-MSCs were exposed to increasing concentrations of HepG2 mitochondria corresponding to 0.025, 0.05, 0.125, and 0.25 mg of proteins per 1 × 10^5^ cells (indicated, respectively, as Mito1, Mito2, Mito3, and Mito4 concentrations) for 24 h in α-MEM. Mito-tracker DEEP Red FM (Beyotime, ShangHai, China, Cat#C1032) was used to label mitochondria and fluorescence tracing sections were captured using a laser scanning confocal microscope (Leica, Wetzlar, Germany).

### 2.3. Electron Microscope Imaging of Isolated HepG2 Mitochondria

The isolated HepG2 mitochondrias were fixed in 2.5% glutaraldehyde at 4°C overnight. After performing a series of standard treatment based protocols, fixed isolated HepG2 mitochondria were cut into ultrathin sections. The microstructure of the copper plated mesh was photographed by ATEM (ThermoFisher's TECNAI spirit 120 kV).

### 2.4. Cell Proliferation

UC-MSCs were exposed to increasing concentrations of HepG2 mitochondria corresponding to 0.025, 0.05, 0.125, and 0.25 mg of proteins per 1 × 10^5^ cells (indicated, respectively, as Mito1, Mito2, Mito3 and Mito4 concentrations) for 24 h in α-MEM. Stained them with Cell Counting Kit-8 reagent kit (CCK-8, Beyotime, ShangHai, China, Cat#C20038) and detect MSC proliferation using a fluorescence enzyme-linked immunosorbent assay.

### 2.5. Experimental Animals

**Figure 1:**
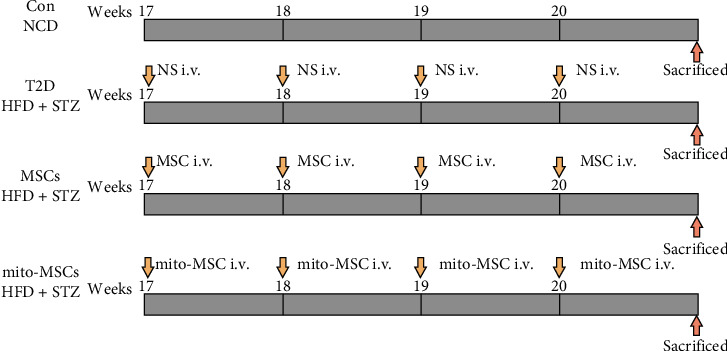
Flowchart of in animal experiment.

Male C57BL/6 mice at the age of 8 weeks were maintained on a free access to standard diet and water in a humidity- and temperature-controlled environment under a 12 h light-dark cycle. The mice of the T2D model group were given high-fat diets for 8 weeks (Sigma-Aldrich, St. Louis, MO, S0130) and then were intraperitoneally injected with 85 mg/kg STZ (Sigma-Aldrich, St. Louis) [28]. The random glucose levels ≥ 16.7 mmol/L for more than 3 days were considered diabetic. The control group (Con) were fed normal chow diets (NCD) for 8 weeks. Mice in T2D groups were infused with 200 uL normal saline through tail vein, and mice in MSCs and mito-MSCs groups were infused with 1 × 10^6^ MSCs suspended in 200 uL normal saline through tail vein, once a week for 4 weeks. The Con, T2D, MSCs, and mito-MSCs group mice were sacrificed 1 week after the last MSCs injection (Figure 1). All the animal experiments complied with the standard ethical guidelines prescribed by the committee of the Chinese PLA General Hospital (Approval Number: SQ2021263).

### 2.6. Western Blot

Mouse liver tissue and HepG2 cells were lysed in lysis buffer, and protein concentration was determined by a BCA Protein Assay Kit (Beyotime, China). Aliquots containing 30 μg of protein were used for western blot analyses, and the experiment was performed following the standard procedure. The primary antibodies were TOM20 (1:1000, rabbit, CST), COXIV (1:1000, mouse, CST), P62 (1:1000, rabbit, CST), and LC3A/B (1:1000, rabbit, CST).The secondary antibodies were goat anti-rabbit (1:3000) and rabbit anti-mouse (1:2500) IgG horse radish peroxidase (HRP, ZSGB-Bio). β-Actin was loaded as an internal control, and the proteins were quantified by the use of Image J software (NIH, Bethesda, MD, USA).

### 2.7. MMP Detection

Cells were digested from the 12-well plate with Trypsin-EDTA (#2360155, Gibco) and washed once with normal saline. 1 × 10^5^ cells were resuspended in 0.5 mL high-glucose DMEM medium. And then dyed through Enhanced mitochondrial membrane potential assay kit with JC-1 (Beyotime, ShangHai, China, Cat#C2003S). 0.5 mL JC-1 staining solution was added, thoroughly mixed, and incubated at 37°C for 20 min in a cell incubator. After incubation, the cells were precipitated by centrifugation at 600 g for 5 min at 4°C. Discard the supernatant and be careful not to lose cells. The cells were washed twice with JC-1 staining buffer (1× ), resuspended with an appropriate amount of JC-1 staining buffer (1× ), and analyzed by Beckman Coulter Epics XL flow cytometer (Beckman Coulter, USA).

### 2.8. Mitochondrial Fluorescence Tracing in Liver Tissue

MSCs/mito-MSCs were stained with Mito tracker DEEP Red FM (Beyotime, ShangHai, China, Cat# C1032) before the last injection. 1 day after injection, collect liver tissue and prepare frozen sections for cell nucleus staining using DAPI. Capture fluorescence tracing sections using laser scanning confocal microscopy (Leica, Wetzlar, Germany).

### 2.9. Blood and Tissue Collection

At the end of the experiment, mice were injected intraperitoneally with 1% pentobarbital sodium (50 mg/kg) anesthesia. Blood was collected and centrifugated at 3000 rpm for 15 min to obtain serum for biochemical analyses. One-third of the fresh liver was rapidly excised and stored at −80°C for mRNA and protein assay. One-fourth of the fresh live was soaked in electron microscope fixative, followed by subsequent sectioning and electron microscopy photography. Then, the mice were perfused through the left ventricle with 10–15 mL PBS, followed by 10–15 mL of 4% paraformaldehyde. After the perfusion, the remaining liver was collected. One-half of the remaining tissue was fixed overnight in 4% paraformaldehyde and then embedded in paraffin to make cross sections of 3 μm thickness for haematoxylineosin (HE). The HE staining was performed to assess hepatic steatosis and inflammatio. Another half of the remaining tissue was incubated in 30% sucrose/PB overnight and then embedded (Tissue-Tek OCT 2 Stem Cells International Compound; Sakura Finetek, Torrance, CA) to make into frozen sections (5 μm) for Oil Red O staining to detect hepatic lipid deposition and for immunofluorescence staining to examine the expression of Mitotracker Red tracing.

### 2.10. Biochemical Analyses

The levels of alanine transaminase (ALT), aspartate aminotransferase (AST), and total cholesterol (TC) in the serum were measured using standard analytics (Beijing Kang Jia Hong Yuan Biological Technology Co. Ltd., China).

### 2.11. Histopathological Examinations and Immunofluorescence Analysis

HE, and Oil Red O were carried out following standard procedures. For immunofluorescence staining, the nuclei of the frozen sections were visualized with 4,6-diamidino-2-phenylindole (DAPI) (Sigma). The HE and Oil Red O staining sections were photographed by a light microscope (Leica TCS SP2, Germany). Evaluation of the extent of NAFLD was performed using the NAFLD activity score (NAS) [29]

### 2.12. HepG2 Mitochondria-Derived ROS Analysis

To determine the ROS levels released by HepG2 mitochondria following their transfer to MSCs, HepG2 mitochondria were isolated from HepG2 cells previously stained with MitoSOX (5 µM, Invitrogen, Waltham, MA, USA, Cat#M36008) for 10 min. MSCs were then exposed to MitoSOX-labeled HepG2 mitochondria for 24 h. MitoSOX fluorescence measurements were carried out with a Tecan Infinite M200 Pro plate reader combined with the acquisition software Magellan 7.2.

### 2.13. Quantitative Real-Time Reverse Transcriptase Polymerase Chain Reaction (qRT-PCR)

Total RNA was extracted from liver tissue, and single-stranded cDNA were synthesized with a reverse transcription kit (Thermo Scientific, CA, USA). Real-time quantitative polymerase chain reaction (PCR) analysis was conducted with a SYBR Green PCR master mix (Applied Biosystems) on ABI Prism thermal cycler model StepOnePus (Applied Biosystems, CA, USA). The thermal cycling program was 95°C for 5 min, followed by 95°C for 15 s, 60°C for 30 s, and 72°C for 30 s for 40 cycles. Melting curve analysis was performed to ensure the specificity of primers. β-actin was used as a reference gene in each sample. The analysis for target gene expression was performed using the relative quantification comparative CT method. The primer sequences used in the qRT-PCR were shown in Table 1.

### 2.14. Statistical Analysis

Data analysis was performed using GraphPad Prism software version 8.0.1(San Diego, CA, USA). Data are expressed as mean ± S.E.M., and for statistical analysis one-way ANOVA combined with Bonferroni multiple comparison tests were applied. *p*-Values smaller than 0.05 were considered significant.

## 3. Results

### 3.1. Characterization of Human UC-MSCs

**Figure 2:**
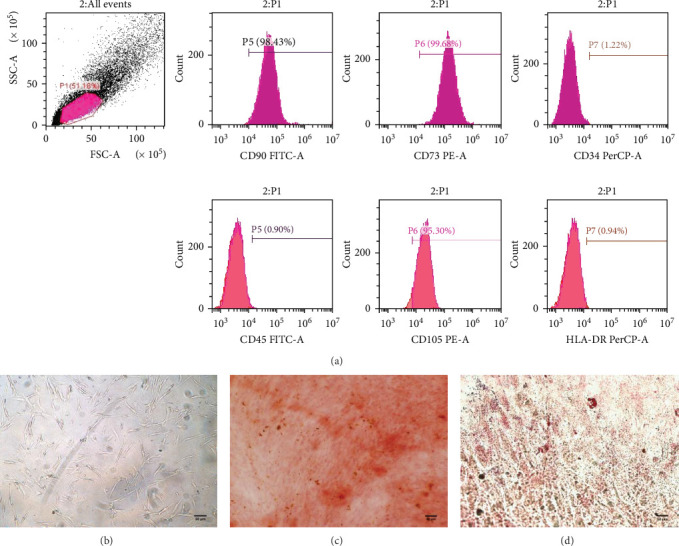
Identification of human UC-MSCs. (a) Flow cytometry analysis of the expression of cell surface markers associated with human UC-MSCs. The expression of each antigen was presented with the corresponding isotype control. (b) Morphological characterization. UC-MSCs are spindle-shaped and fibroblast-like. Scale bar = 100 μm. (c) Alizarin red S staining of cultured osteogenic human UC-MSCs. Scale bar = 100 μm. (d) Oil red O staining of cultured lipogenic human UC-MSCs. Scale bar = 50 μm.

To further identify the adherent cells, immunophenotypic. features and multilineage differentiation potential were examined. As presented in Figure 2a, the cells expressed surface marker characteristic of UC-MSCs, including CD90, CD73, and CD105, while negative surface markers of UC-MSCs, including CD34, CD45, and HLA-DR, were not expressed. The cultured human UC-MSCs have a bipolar spindle-like and fibroblastoid-shaped morphology (Figure 2b). Moreover UC-MSCs exhibited potential to differentiate into osteoblasts (Figure 2c) and adipocytes (Figure 2d).

### 3.2. Detection of Mitochondrial Function and Proliferative Aspects of MSC After Pretreatment of MSC With Different Concentrations of Mitochondria

**Figure 3:**
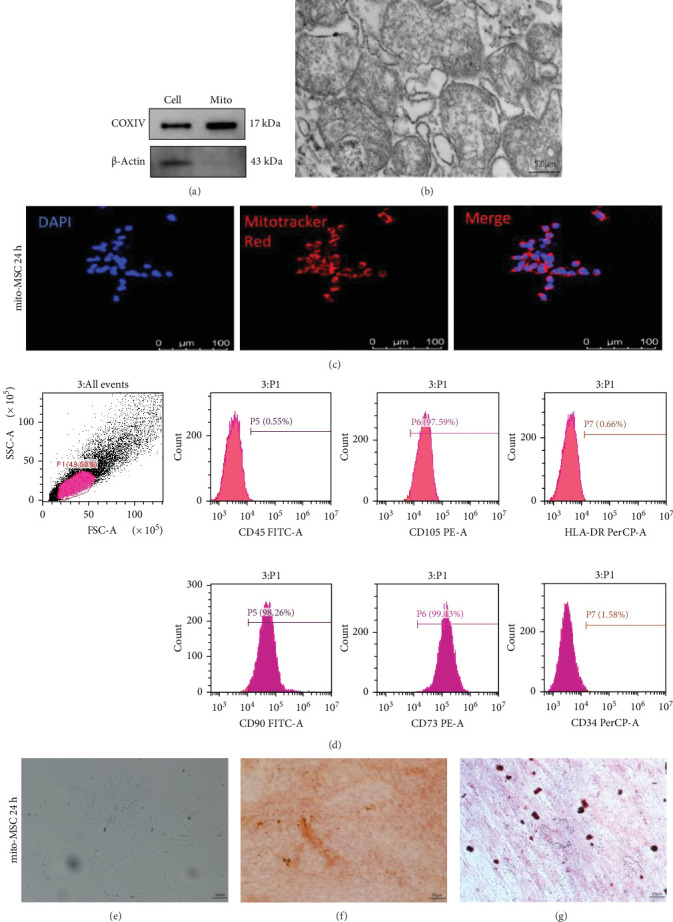
Identification of free mitochondria extraction and UC-MSCs pretreatment for 24 h. (a) Protein blotting to assess the protein level of COXIV and β-actin of free mitochondria and total protein from HepG2 cells. (b) Morphology of free mitochondria under electron microscope. Scale bar = 5 μm. (c) Mitotracker fluorescence tracing showing the state of free mitochondria into MSCs 24 h (red fluorescence. Scale bar = 100 μm). (d) Flow cytometry analysis of the expression of cell-surface markers associated with human UC-MSCs after free mitochondria preconditioning with UC-MSCs 24 h. The expression of each antigen was presented with the corresponding isotype control. (e) Morphological characterization. MSCs are spindle-shaped and fibroblast-like. Scale bar = 50 μm. (f) Alizarin red S staining of cultured osteogenic human UC-MSCs. Scale bar = 50 μm. (g) Oil red O staining of cultured lipogenic human UC-MSCs. Scale bar = 50 μm.

Free mitochondrial suspension extracted from HepG2 was incubated at 37°C, 5% CO_2_ incubator for 3 days and no adhesion of HepG2 cells was detected. By comparing the extracted free mitochondrial proteins with the mitochondrial protein marker COXIV and cytoskeletal protein marker β-actin of total cellular proteins by WB assay (Figure 3a) as well as by downstream observation of the morphology of the free mitochondria by electron microscopy (Figure 3b), we confirmed that the extracted mitochondria were indeed free mitochondria. After coculturing free mitochondria labeled with Mitotracker Red with UC-MSCs for 24 h, observations were made by confocal microscopy to confirm that the free mitochondria had entered the UC-MSCs (Figure 3c). After free mitochondria pretreated UC-MSCs for 24 h, the expression of cell surface markers associated with human UC-MSCs (Figure 3d), morphological characteristics (Figure 3e) and potential to differentiate into osteoblasts (Figure 3f) and lipogenic cells (Figure 3g) were identified by flow cytometry. We determined that pretreatment of UC-MSCs with mitochondria from HepG2 cells did not result in changes in the properties of UC-MSCs.

### 3.3. Screening of Mitochondrial Pretreated UC-MSCs at Gradient Concentrations and Alterations in MSC Proliferation and Mitochondrial Function

**Figure 4:**
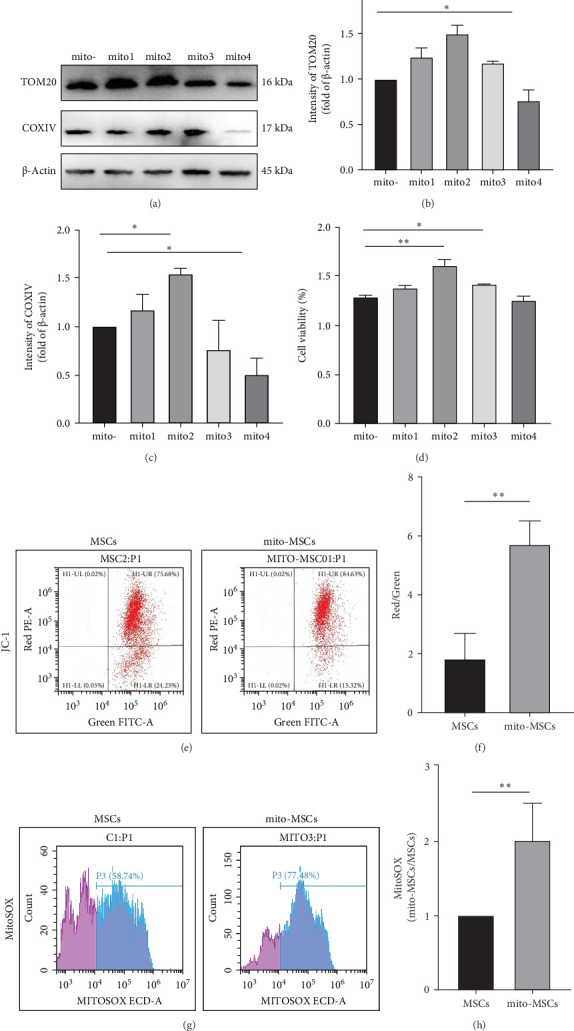
Concentration screening of mitochondria-treated MSCs after 24 h and changes in MSCs mitochondrial function after pretreatment. (a) Protein blotting to assess the protein level of TOM20 and COXIV after different concentrations of free mitochondria treatment of UC-MSCs for 24 h. (b) TOM20 protein level. (c) COXIV protein level. (d) CCK8 assay to assess the level of MSCs proliferation after different concentrations of free mitochondria treated UC-MSCs for 24 h. (e, f) Flow cytometry assessment and statistical analysis of JC-1 levels in MSCs before and after pretreatment. (g, h) Flow cytometry assessment and statistical analysis of MitoSOX levels in MSCs before and after mitochondrial pretreatment of MSCs. (*n* = 3) Each experiment was repeated three times and typical pictures are shown. Data are expressed as mean ± SD. *⁣*^*∗*^*p* < 0.05; *⁣*^*∗∗*^*p* < 0.01.

We pretreated MSCs with exogenous mitochondria sourced from various origins and exhibiting different functional states, and found no statistically significant differences in the expression levels of mitochondrial marker proteins among the various groups of MSCs (Supporting Information 1: Figure S1). HepG2-derived free mitochondria were pretreated with mito1 (0.025 mg), mito2 (0.05 mg), mito3 (0.125 mg), and mito4 (0.25 mg) mitochondrial protein amounts, respectively, in 1 × 10^5^ MSCs for 24 h. By western blot experiments (Figure 4a–c) and CCK-8 (Figure 4d), we used mito2 as the final concentration acting on UC-MSCs, labeled as mito-MSCs group. Then we detected JC-1 and MitoSOX in free mitochondria pretreated MSCs by flow-through assay (Figure 4e–h), and found that the MMP and mitochondrial ROS in the mito-MSCs group were significantly increased compared with the untreated MSCs group. The above results indicated that pretreatment of mitochondria could enhance the mitochondrial function and proliferation ability of MSCs.

### 3.4. Improvement in Blood Glucose, Body Weight, and Liver Function and Steatosis After Pretreatment With MSC Administered via Tail Vein Injection to C57 Diabetes Model Mice

**Figure 5:**
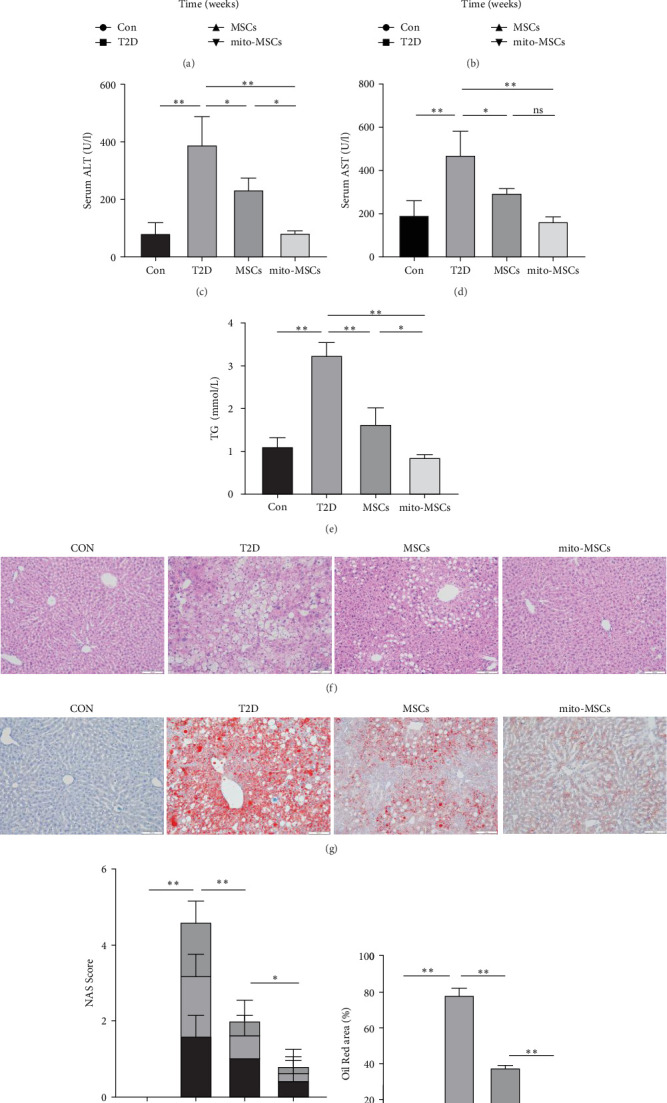
Mitochondria-pretreated human UC-MSCs infusion enhances the effect of MSCs in alleviating functional liver injury and improves blood glucose, liver function, and hepatic steatosis. Human MSCs, mito-MSCs, or normal saline was infused to C57 mice fed high-fat for 8 weeks + STZ once a week for 4 weeks. Fasting blood glucose (a), body weight (b), serum ALT (c), AST (d), TG (e) after 4 weeks of infusion (f). Representative image panel of HE-stained sections of liver tissue. (Scale bar = 50 μm). (g) Representative images of oil red O-stained liver sections of the indicated groups. Scale bar = 50 μm. (h) Scoring of NAS in the indicated groups, *n* = 5 sections per group. (i) Quantitative analysis of oil red O-positive areas in the region, *n* = 5 sections per group. All data are expressed as mean ± SD. Each group *n* = 4 mice. *⁣*^*∗*^*p* < 0.05; *⁣*^*∗∗*^*p* < 0.01.

According to previous literature, 8-week-old C57 mice were injected with 100 mg/kg STZ after 8 weeks of HFD feeding. random blood glucose levels ≥ 16.7 mmol/L for three consecutive days were considered successful for diabetic model construction (T2D mice). UC-MSCs/mito-MSCs/normal saline were injected into T2D mice via tail vein once a week for four consecutive weeks. Mouse body weight measurements and fasting blood glucose measurements were performed 1 day before each injection. At baseline, blood glucose and body weight of T2D mice on HFD + STZ increased dramatically compared to C57 mice on regular diet (Con). Treatment with UC-MSCs (MSCs group) was able to significantly reduce blood glucose and body weight in T2D mice. Mito-MSCs treatment (mito-MSCs group) was able to promote its blood glucose lowering effect more significantly compared to the MSCs group, but there was no significant improvement in body weight lowering compared to the MSCs group (Figure 5a,b). Then we assessed functional liver injury by measuring serum ALT and AST levels. The results showed a sharp increase in both ALT and AST in the T2D group compared with the Con, indicating that the liver function of T2D mice was severely impaired. After treatment with UC-MSCs, both ALT and AST in the MSCs group showed significant decreased (Figure 5c,d). Compared with MSCs, ALT decreased significantly in the mito-MSCs group; AST showed a decreasing trend, but there was no significant difference. Abnormal blood lipids and hepatic lipid deposition are important features of NAFLD. Our results showed that the T2D group showed a significant increase in TG, while the treatment of UC-MSC reduced the level of TG in diabetic mice. A significant decrease in TG levels was also observed in mito-MSCs compared to the MSCs group (Figure 5e). Occasional inflammation was observed in the liver of the T2D group. In contrast, the MSCs group showed reduced hepatocyte volume, fewer intracellular lipid droplets, and reduced ballooning injury. In contrast, the therapeutic effect of UC-MSCs on hepatocytes was significantly enhanced in the mito-MSCs group. Compared with the T2D group, there was no significant change in liver inflammation in the MSCs group, but a more significant decrease was observed in the mito-MSCs group (Figure 5f,h). In addition, oil red O-positive areas were significantly increased in T2D mice and significantly decreased in MSC-treated mice. And a significant decrease in the percentage of oil red O-positive regions was also observed in the mito-MSCs group compared to the MSCs group (Figure 5g,i). In conclusion, these results suggest that infusion of free mitochondria-pretreated UC-MSCs enhanced the therapeutic effects of MSCs on blood glucose, lipids, hepatic steatosis and lipid accumulation in T2D mice.

### 3.5. Free Mitochondria Preconditioned UC-MSCs Enhanced the Ameliorative Effect of UC-MSCs on NAFLD in Type 2 Diabetic Mice by Promoting Mitochondrial Translocation of Mitochondria, Which May Be Related to the Ability of Free Mitochondria to Trigger Autophagy in UC-MSCs

**Figure 6:**
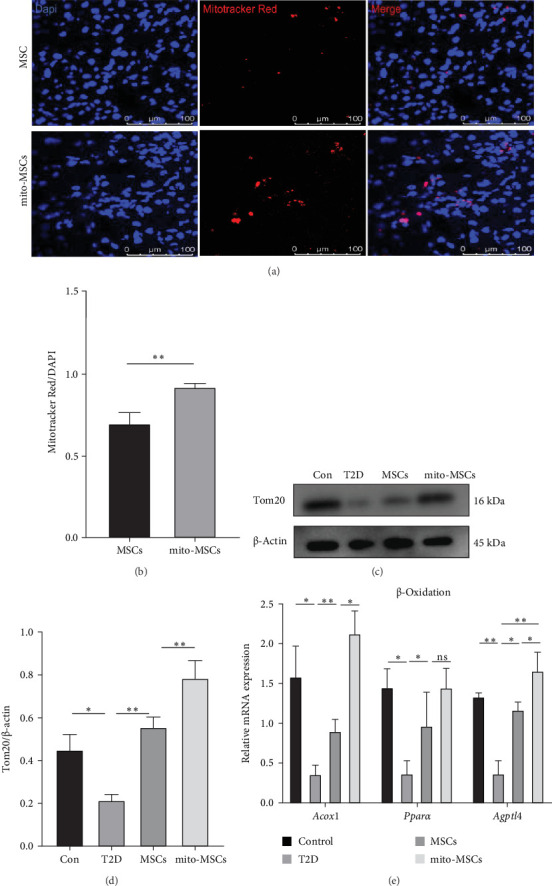
Mitochondrial pretreatment increased the transport of mitochondria from MSCs to damaged liver cells, improving mitochondrial function and β-oxidation in T2D mouse liver cells. (a) Immunofluorescence was used to assess the number of MSCs mitochondria entering hepatocytes 24 h after tail vein injection of MSCs before and after pretreatment. (b) Quantification of the ratio of mitochondrial fluorescence tracer intensity. (c) WB assessment of Tom20 expression level in hepatocytes 4 weeks after pretreatment of MSC injected into T2D mice. (d) Statistical analysis of Tom20 protein levels. (e) qRT-PCR assessment of hepatic tissue β-oxidation levels in pretreated MSC-injected T2D mice after 4 weeks. Data are expressed as mean ± SD. *⁣*^*∗*^*p* < 0.05; *⁣*^*∗∗*^*p* < 0.01.

**Figure 7:**
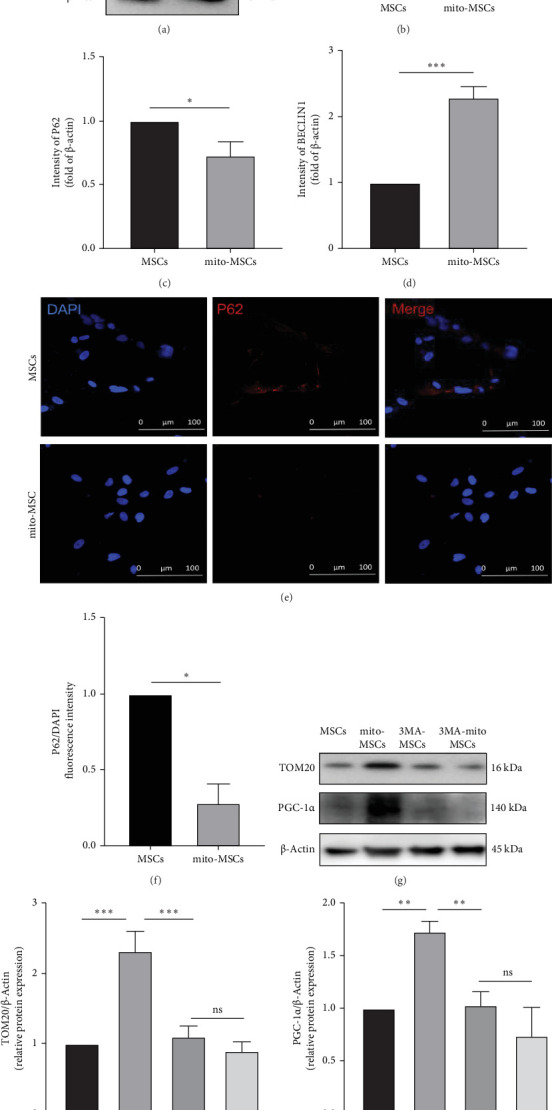
The enhanced effect of mitochondrial pretreatment on NAFLD in T2D mice treated with MSCs may be related to its promotion of MSCs autophagy and mitochondrial generation. (a) Protein blotting to assess the protein level of LC3A/B, P62, and BECLIN1 protein levels before and after pretreatment of UC-MSCs with free mitochondria. (b–d) Statistical analysis of LC3A/B, P62 and BECLIN1 levels in MSC before and after pretreatment. (e, f) The P62 fluorescence intensity and statistical analysis of MSCs cells before and after pretreatment showed by confocal images; Red: P62; Blue: nucleus. (g) Protein blotting to assess the protein level of TOM20 and PGC-1α protein levels before and after pretreatment of UC-MSCs with free mitochondria and 3-MA. (h, i) Statistical analysis of TOM20 and PGC-1α levels in MSC before and after pretreatment. Each experiment was repeated three times and typical pictures are shown. Data are expressed as mean ± SD. *⁣*^*∗*^*p* < 0.05; *⁣*^*∗∗*^*p* < 0.01; *⁣*^*∗∗∗*^*p* < 0.001.

By prestaining the mitochondria of MSCs/mito-MSCs injected in the tail vein, mice were executed 24 h after injection and liver sampling and immunofluorescence were evaluated, which revealed a significant increase in the fluorescence intensity of mitotracker Red in the mito-MSCs group in comparison with the MSCs group (Figure 6a,b). Analysis of the immunoprotein blotting of fresh liver tissues from mice in each group showed that the expression of Tom20 in the MSCs group presented a significant increase compared to the significant decrease in Tom20 in the T2D group. And the expression of Tom20 in the mito-MSCs group had a more significant increase graph compared to the MSCs group (Figure 6c,d). This demonstrated that free mitochondria preconditioned MSCs could enhance the rescue of UC-MSCs for the injured liver mitochondrial function in T2D. Next, we assessed the transcript levels of genes related to fatty acid β-oxidation (*Pparα* and its target genes *Acox1* and *Angplt4*) in the liver tissues of each group of mice by qRT-PCR, and found that all genes related to β-oxidation were significantly decreased in T2D mice, and significantly upregulated after infusion of UC-MSCs, whereas free mitochondrial preconditioning more significantly elevated the upregulation of β-oxidation-related genes in liver tissue by UC-MSCs (Figure 6e).We have also proven this in vitro (Supporting Information 2: Figure S2). Finally, as previous literature showed that damps entering MSCs may trigger their autophagy, we evaluated the autophagy level of MSCs before and after free mitochondria pretreatment by Western blot analysis and confocal immunofluorescence (Figure 7a–f) and found that the autophagy level of mitochondria was significantly elevated after pretreatment. After blocking autophagy by 3-MA, we verified the expression of TOM20 and PGC-1α in MSCs before and after pretreatment, and found that the enhancing effect of pretreatment was significantly reduced with autophagy blocked (Figure 7g–i).Upon inhibiting or enhancing the autophagy of MSCs, the quantity of mitochondria transferred from MSCs pretreated with exogenous mitochondria to injured HepG2 cells decreased or increased correspondingly (Supporting Information 3: Figure S3). We also explored the changes of PINK1/PARKIN, a classical mitophagy pathway, during pretreatment (Supporting Information 4: Figure S4). Therefore, we hypothesized that the entry of free mitochondria into MSCs triggered mitochondrial autophagy in MSCs, elevated the number and function of mitochondria in MSCs, which in turn prompted the transfer of more mitochondria in MSCs to damaged hepatocytes, and ultimately facilitated the transcription of genes related to fatty acid β oxidation in hepatocytes.

## 4. Discussion

In this study, we used exogenous liver mitochondria to pretreat UC-MSCs, and it was confirmed that compared with UC-MSCs, pretreatment of UC-MSCs with exogenous mitochondria could enhance therapeutic effect in reducing blood glucose, transaminase, and blood lipid levels, improving lipid deposition and liver injury of NAFLD.

Human umbilical cord MSCs, as an important cell source in the field of regenerative medicine, have distinct tissue distribution characteristics. These stem cells are mainly isolated from umbilical cord blood, Wharton's jelly, and the peripheral stroma of umbilical vessels and other umbilical cord-related tissues [30]. Notably, compared with bone marrow-derived MSCs (BM-MSCs) and adipose-derived MSCs (AD-MSCs), hUC-MSCs exhibit more significant proliferation kinetics in vitro. UC-MSCs do not express MHC-II, have very low immunogenicity, and show immunomodulatory properties in vivo, making them excellent substitutes for allogeneic and xenogeneic transplantation in cell therapy [31]. UC-MSCs can improve diseases and clinical symptoms, such as Parkinson's disease, tissue fibrosis, cancer, and spinal cord injury through their multidirectional differentiation potential and unique paracrine mechanism [32]. Previous studies from our research group have also shown that they have significant therapeutic effects in treating T2D and related complications.

Mitochondrial transfer has been demonstrated as a crucial therapeutic mechanism of MSCs. Studies have revealed that MSCs derived from various tissues exhibit mitochondrial transfer during the treatment of cardiac, pulmonary, hepatic, and respiratory smooth muscle injuries [7, 33–36]. Previous literature has indicated that following injection into T2D mice via the tail vein, MSCs can migrate abundantly to the lung, spleen, and liver [37]; thus highlighting their potential efficacy [38] in treating liver diseases, such as NAFLD. Thus, the situation unfolded as described, Bi et al. [7] investigation on bone marrow-derived MSC therapy for NAFLD-induced hepatic injury demonstrated that BM-MSC implantation effectively mitigated high-fat diet-induced steatosis while significantly restoring liver function and ameliorating glucose and lipid metabolism disorders in T2DM-associated NAFLD mice. These beneficial effects were accompanied by a reduction in fat accumulation. Compared with steatosis, the pretreatment group in this study showed a trend but not significant improvement in the therapeutic function of MSCs in terms of swelling and inflammation. This might be related to the fact that MSCs themselves have powerful anti-inflammatory and regenerative functions [39, 40]. The MSCs group had already nearly reversed the swelling and inflammation in T2D mice, so there were no more significant changes before and after pretreating the MSCs. However, the liver has a rich content of mitochondria, and mitochondrial dysfunction is one of the important mechanisms of abnormal lipid deposition in the liver [41]. Our pretreatment method has more specifically improved the mitochondrial function of hepatocytes, and therefore has a more significant effect in improving steatosis. Given the abundance of mitochondria in the liver and their crucial role in NFALD, mitochondrial transfer may hold greater significance in treating NAFLD with MSCs. Notably, both in vivo and in vitro studies on BM-MSCs for T2D-related NAFLD have observed mitochondrial transfer [7]. Invitro experiments specifically demonstrated that hepatocytes with liver steatosis exhibited significantly improved OXPHOS activity, ATP production, mitochondrial membrane potential, and reduced reactive oxygen levels upon receiving healthy mitochondria from MSCs. These findings indicate a substantial amelioration of mitochondrial dysfunction in T2D-related NAFLD mice through mitochondrial transfer. Our results corroborate these observations as UC-MSCs treatment led to decreased blood glucose levels, liver transaminase levels, triglyceride levels, mitigated histological damage, and confirmed similar occurrences of mitochondrial transfer.

As a type of DAMPs, free mitochondria can augment the therapeutic efficacy of MSCs towards injured target cells. Our study further substantiated these findings, as treatment with UC-MSCs cocultured with HepG2-derived mitochondria in T2D mice for 4 weeks significantly attenuated hepatic lipid accumulation, reduced fasting blood glucose levels and liver enzymes ALT, AST, and TG compared to pretreatment conditions. These results indicate that exogenous mitochondria effectively enhance the therapeutic potential of MSCs against liver injury. Liver free fatty acids (FFAs) originate from plasma FFAs released by adipose tissue and intestinal intraperitoneal granules or are synthesized de novo within hepatocytes [42]. These FFAs are either directed into the mitochondria for β-oxidation or esterified and stored as triglycerides [43]. Consequently, impaired β-oxidation capacity during mitochondrial dysfunction promotes hepatic lipid accumulation. Our findings revealed increased expression levels of β -oxidation-related genes (ACOX, PPARa, Angptl4, and cpt1b) in liver tissue treated with UC-MSCs pretreated with exogenous mitochondria, indicating that pretreated UC-MSCs enhance the restorative ability of MSCs to promote β-oxidation in NAFLD, thereby improving liver function and mitigating liver tissue injury.

Since mitochondrial pretreatment of UC-MSCs did not alter the biological characteristics of UC-MSCs, we also observed that UC-MSCs could uptake exogenous mitochondria after coculture. Therefore, we hypothesized that exogenous mitochondria as damage-associated molecular patterns (DAMPs) could upregulate the quantity and functionality of mitochondria in UC-MSCs themselves, which was further confirmed by experimental results. Furthermore, Mahrouf-Yorgov et al. [25] have substantiated that exogenous mitochondria augment the capacity of MSCs to transfer mitochondria to impaired cells through coculturing MSCs with damaged cardiomyocytes and endothelial cells. The activation of MSCs is driven by exogenous ROS-dependent mitophagy, which stimulates the cytoprotective enzyme HO-1 and induces mitochondrial biomass in MSCs [25]. Vignais et al. [44]also observed that myocardial-derived mitochondria induceself-degradation in MSCs by generating ROS as a mediator for stimulation. Our study additionally revealed that after 24 h of pretreatment with free mitochondria from HepG2, mtROS levels were significantly enhanced in UC-MSCs, accompanied by significant upregulation of mitophagy-related proteins. These findings suggest that HepG2-derived mitochondria may facilitate mitochondrial transfer from UC-MSCs to damaged liver tissue by triggering autophagy.

Although we have confirmed that exogenous mitochondria can facilitate the transfer of healthy mitochondria from UC-MSCs to injured liver cells, and identified a potential association with the activation of autophagy in MSCs by exogenous mitochondria, thereby promoting generation and transfer of mitochondria, ultimately elevating β-oxidation levels in liver tissue for lipid metabolism regulation. Nonetheless, further investigations are warranted to elucidate the mechanisms underlying the entry of exogenous mitochondria into MSCs and their subsequent induction of regulatory autophagy. In any case, our study presents a novel therapeutic approach for enhancing the efficacy of MSC-based interventions against T2D-induced hepatic injury in NAFLD.

## Figures and Tables

**Table 1:** The primer sequences used for the q-PCR.

Gene name	Forward (5′–3′)	Reverse (5′–3′)
*β-Actin*	CATTGCTGACAGGATGCAGAAGG	TGCTGGAAGGTGGACAGTGAGG
*Acox1*	AGGGAATTTGGCATCGCAG	GATTCAGCAAGGTAGGGATAAACA
*Pparα*	TTTCACAAGTGCCTGTCTGTCG	TCTTCAGGTAGGCTTCGTGGAT
*Angptl4*	GGATAGAGTCCCTGAAGGCCA	TGAGCTGGGTCATCTTGGGA

## Data Availability

The data sharing is not applicable to this article as no datasets were generated or analyzed during the current study.

## Supporting Information

Supporting Information 1 Figure S1. Changes in mitochondrial marker protein expression after pretreating MSCs with exogenous mitochondria from different origins and functional states. (a) Protein blotting to assess the protein level of TOM20 and COXIV after treating UC-MSCs with HepG2, mc3t3, and free mitochondria derived from HepG2 treated with ethidium bromide for 24 h. (b) TOM20 protein level. (c) COXIV protein level. Each experiment was repeated three times and typical pictures are shown. Data are expressed as mean ± SD. ⁣^*∗*^*p* < 0.05.

Supporting Information 2 Figure S2. Fatty acid oxidation rate in HepG2 cells damaged by high glucose before and after exogenous mitochondrial pretreatment. Each experiment was repeated three times. Data are expressed as mean ± SD. ⁣^*∗*^*p* < 0.05; ⁣^*∗∗*^*p* < 0.01; ⁣^*∗∗∗*^*p* < 0.001.

Supporting Information 3 Figure S3. Following the inhibition or enhancement of autophagy in MSCs, the mitochondrial transfer from MSCs to damaged HepG2 cells was observed before and after pretreatment. Immunofluorescence and fluorescence intensity analysis of mitochondria (Mitotracker Red) in damaged HepG2 cells were conducted after downregulating (CQ-mito-MSCs) or upregulating (RAPA-mito-MSCs) the autophagy function in MSCs. Each experiment was repeated three times and typical pictures are shown. Data are expressed as mean ± SD. ⁣^*∗*^*p* < 0.05; ⁣^*∗∗*^*p* < 0.01; ⁣^*∗∗∗*^*p* < 0.001.

Supporting Information 4 Figure S4. (a) After the autophagy of UC-MSCs was blocked, the expression of mitophagy proteins PINK1 and PARKIN in MSC before and after Mito pretreatment. (b,c) The protein expression of PINK1, PARKIN, and gray analysis was detected by Western blot. (d) Fluorescence microscopy showed that the mitochondrial marker Mitotracker Green and lysosome marker LysoTracker Red colocalized in MSCs before and after pretreatment; Red: lysosomes; Green: mitochondria; Blue: nucleus.*⁣*^*∗*^*p* < 0.05, *⁣*^*∗∗*^*p* < 0.01, *⁣*^*∗∗∗*^*p* < 0.001, *⁣*^*∗∗∗∗*^*p* < 0.0001, NS is not statistically significant.
