# Supplementary figures and images for: Exogenous Mitochondrial Pretreatment Enhances the Therapeutic Effect of UC-MSCs on NAFLD in Type 2 Diabetic Mice by Mediating Mitochondrial Transfer
Source: Stem Cells Int. 2025 Aug 25;2025:4639115. doi: 10.1155/sci/4639115 (PMC12401616; doi:10.1155/sci/4639115)

## Slide 1
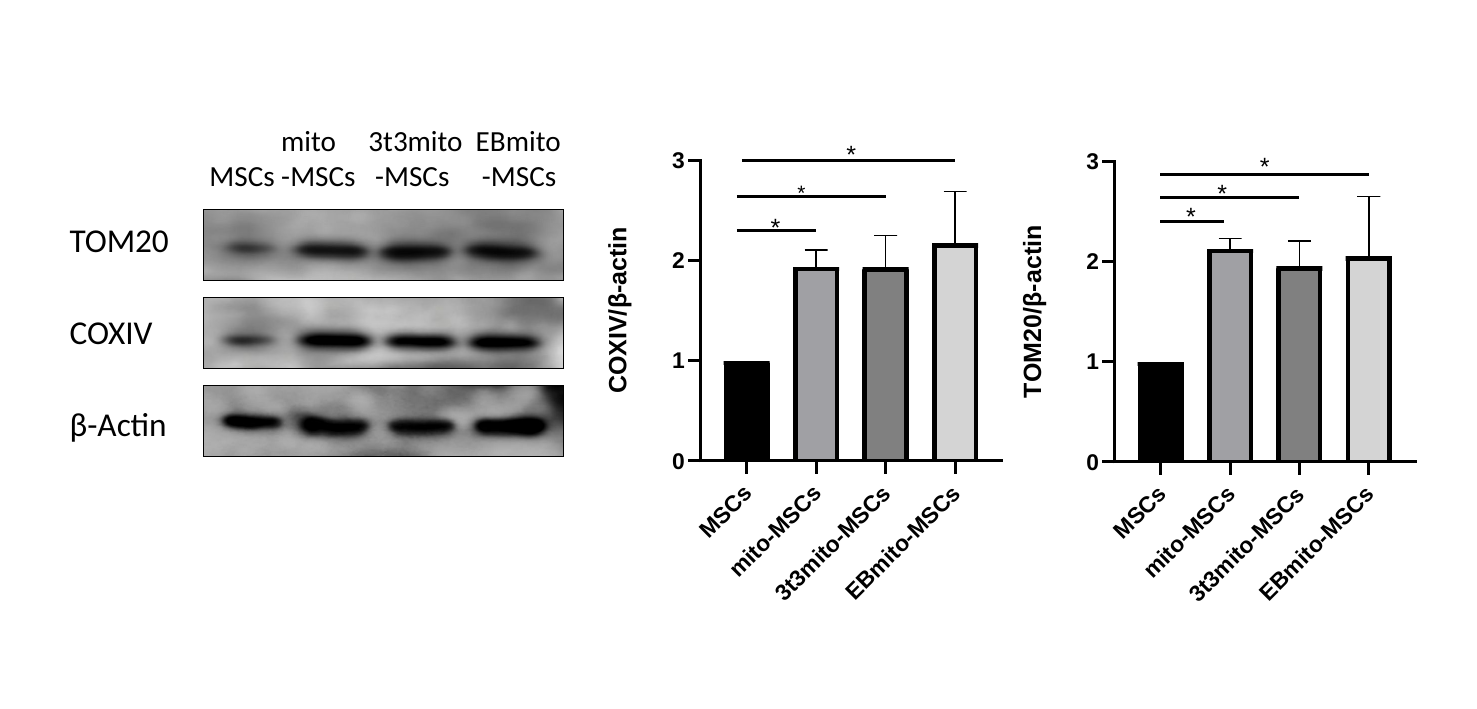

mito 3t3mito EBmito
MSCs -MSCs -MSCs -MSCs
TOM20
COXIV
β-Actin

Supplement: Supporting Information 1 — Figure S1. Changes in mitochondrial marker protein expression after pretreating MSCs with exogenous mitochondria from different origins and functional states. (a) Protein blotting to assess the protein level of TOM20 and COXIV after treating UC-MSCs with HepG2, mc3t3, and free mitochondria derived from HepG2 treated with ethidium bromide for 24 h. (b) TOM20 protein level. (c) COXIV protein level. Each experiment was repeated three times and typical pictures are shown. Data are expressed as mean ± SD. ⁣∗p < 0.05. [file 4639115.f1.pptx]

## Slide 1
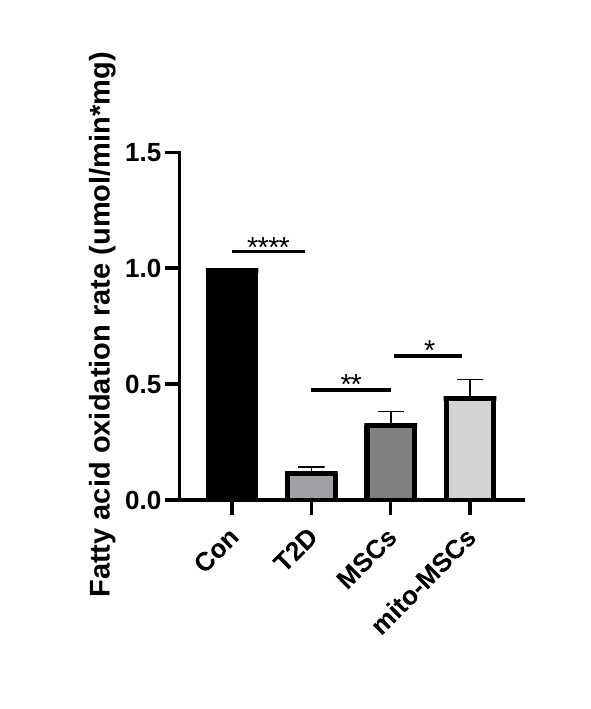

Supplement: Supporting Information 2 — Figure S2. Fatty acid oxidation rate in HepG2 cells damaged by high glucose before and after exogenous mitochondrial pretreatment. Each experiment was repeated three times. Data are expressed as mean ± SD. ⁣∗p < 0.05; ⁣∗∗p < 0.01; ⁣∗∗∗p < 0.001. [file 4639115.f2.pptx]

## Slide 1
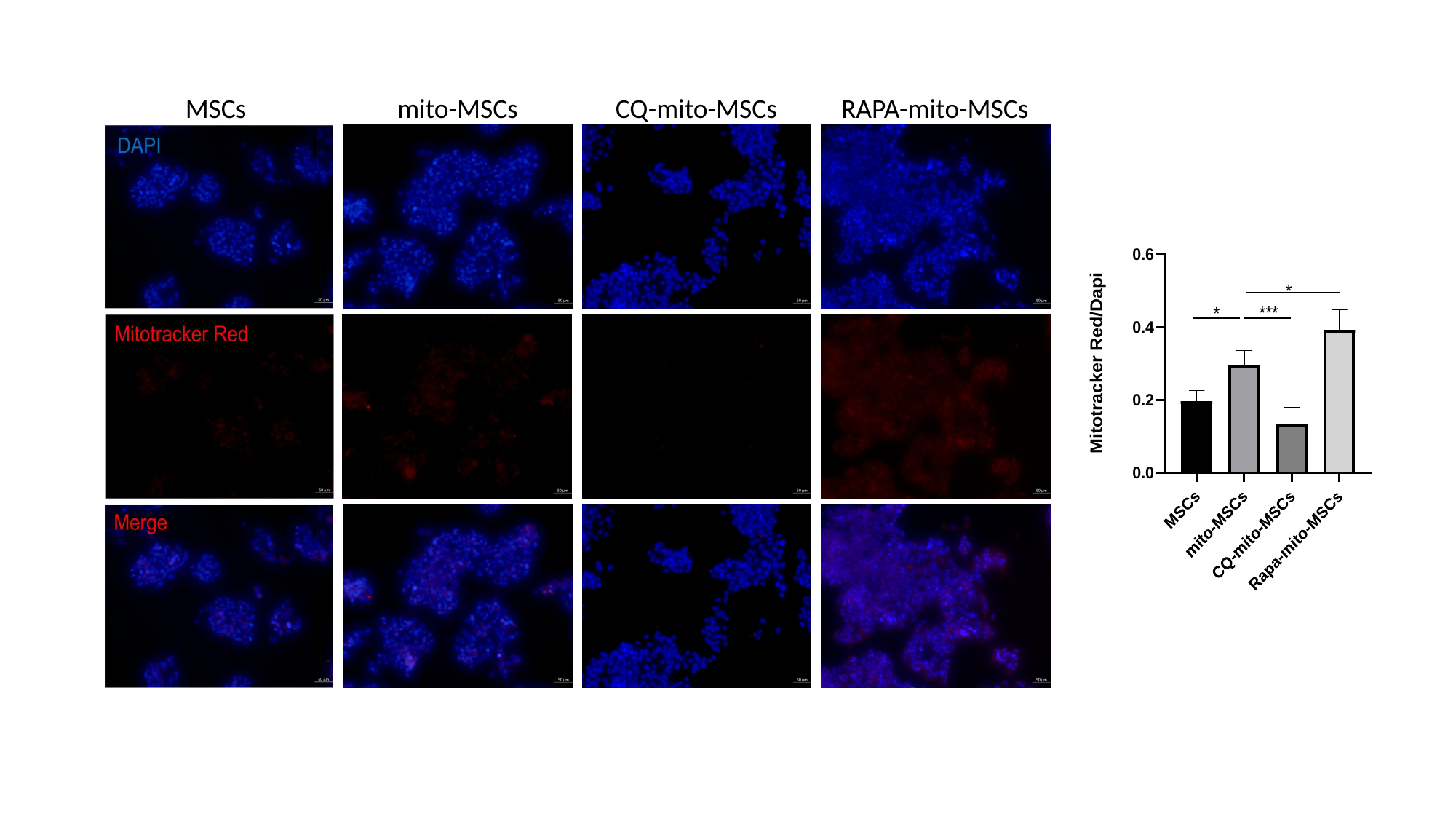

MSCs
mito-MSCs
RAPA-mito-MSCs
CQ-mito-MSCs

Supplement: Supporting Information 3 — Figure S3. Following the inhibition or enhancement of autophagy in MSCs, the mitochondrial transfer from MSCs to damaged HepG2 cells was observed before and after pretreatment. Immunofluorescence and fluorescence intensity analysis of mitochondria (Mitotracker Red) in damaged HepG2 cells were conducted after downregulating (CQ-mito-MSCs) or upregulating (RAPA-mito-MSCs) the autophagy function in MSCs. Each experiment was repeated three times and typical pictures are shown. Data are expressed as mean ± SD. ⁣∗p < 0.05; ⁣∗∗p < 0.01; ⁣∗∗∗p < 0.001. [file 4639115.f3.pptx]
