# Supplementary material for: Exogenous Mitochondrial Pretreatment Enhances the Therapeutic Effect of UC-MSCs on NAFLD in Type 2 Diabetic Mice by Mediating Mitochondrial Transfer
Source: Stem Cells Int. 2025 Aug 25;2025:4639115. doi: 10.1155/sci/4639115 (PMC12401616; doi:10.1155/sci/4639115)
Supplement: Supporting Information 4 — Figure S4. (a) After the autophagy of UC-MSCs was blocked, the expression of mitophagy proteins PINK1 and PARKIN in MSC before and after Mito pretreatment. (b,c) The protein expression of PINK1, PARKIN, and gray analysis was detected by Western blot. (d) Fluorescence microscopy showed that the mitochondrial marker Mitotracker Green and lysosome marker LysoTracker Red colocalized in MSCs before and after pretreatment; Red: lysosomes; Green: mitochondria; Blue: nucleus.⁣∗p < 0.05, ⁣∗∗p < 0.01, ⁣∗∗∗p < 0.001, ⁣∗∗∗∗p < 0.0001, NS is not statistically significant. [file 4639115.f4.pptx]

## Slide 1
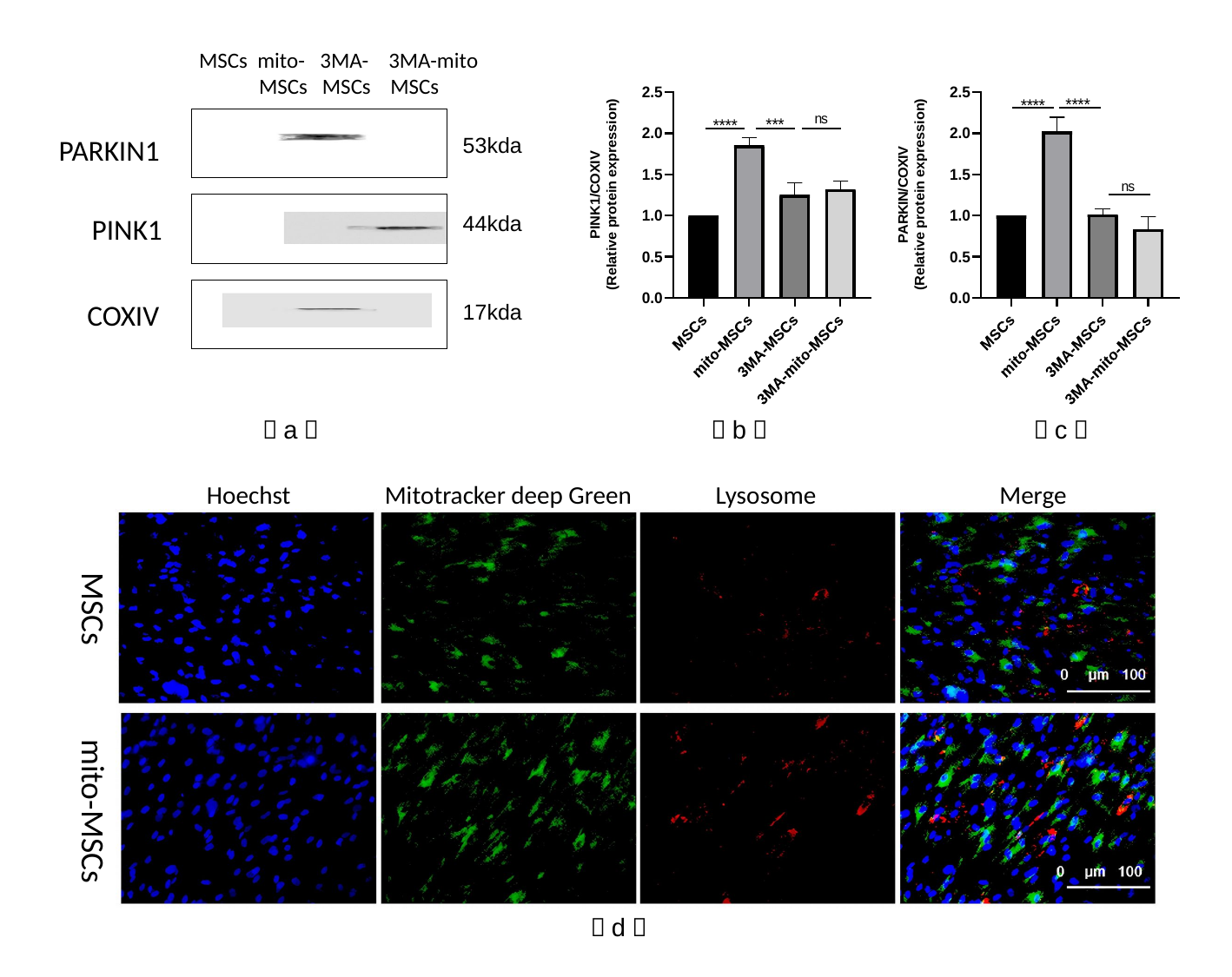

MSCs mito- 3MA- 3MA-mito
 MSCs MSCs MSCs
PARKIN1
53kda
44kda
PINK1
COXIV
17kda
（c）
（d）
（a）
（b）
Hoechst
Mitotracker deep Green
Lysosome
Merge
MSCs
mito-MSCs
